# Beneficiários de planos privados de saúde que utilizaram a rede pública de hemodiálise ambulatorial no Brasil entre 2012 e 2019 

**DOI:** 10.1590/0102-311XPT188422

**Published:** 2023-10-09

**Authors:** Laires Cristina Amorim, Mariangela Leal Cherchiglia, Ilka Afonso Reis

**Affiliations:** 1 Faculdade de Medicina, Universidade Federal de Minas Gerais, Belo Horizonte, Brasil.; 2 Instituto de Ciências Exatas, Universidade Federal de Minas Gerais, Belo Horizonte, Brasil.

**Keywords:** Compensação e Reparação, Hemodiálise, Saúde Suplementar, Sistema Único de Saúde, Compensation and Redress, Hemodialyses, Supplemental Health, Unified Health System, Compensación y Reparación, Hemodiálisis, Salud Complementaria, Sistema Único de Salud

## Abstract

O ressarcimento ao Sistema Único de Saúde (SUS) é a interface mais visível da relação entre saúde pública e privada, e sua análise pode ampliar o conhecimento sobre o uso do SUS pelo setor suplementar. O presente estudo objetivou caracterizar os beneficiários de planos privados de saúde que realizaram hemodiálise no SUS entre 2012 e 2019 em relação a: sexo, faixa etária, região de residência, características dos planos privados de saúde e das operadoras e a assistência prestada a eles. Visou também comparar características dos planos privados de saúde e modalidade das operadoras daqueles beneficiários com dados dos demais beneficiários do Brasil. Construiu-se uma base centrada no indivíduo a partir de dados da Agência Nacional de Saúde Suplementar (ANS); informações sobre beneficiários do Brasil foram consultadas no Departamento de Informática do SUS (DATASUS). Utilizou-se distribuições de frequências para resumir os dados, padronização por idade e sexo para características dos planos privados de saúde e modalidade das operadoras, e razão para comparar frequências. Um total de 31.941 beneficiários realizou hemodiálise no SUS, 11.147 (34,9%) destes fora de seu município de residência, e 6.423 (20,11%) utilizaram o SUS por 25 meses ou mais. Comparados aos demais beneficiários do Brasil, aqueles que realizaram hemodiálise no SUS estavam vinculados mais frequentemente a planos privados de saúde antigos (razão, r = 2,41), coletivos por adesão (r = 1,76), individuais/familiares (r = 1,36), ambulatoriais (r = 4,66), municipais (r = 3,88) e/ou a filantropias (r = 7,32). Planos privados de saúde com características restritivas podem ter dificultado o acesso dos beneficiários que realizaram hemodiálise no SUS às redes de suas operadoras, e representado mais um fator que pode ter influenciado o uso do SUS por aqueles beneficiários, mesmo com a cobertura prevista em seus contratos.

## Abstract (en)

Reimbursement to Brazilian Unified National Health System (SUS) is the most visible interface of the public-private relationship and its analysis can expand our understanding of the use of SUS by the supplementary sector. The study aims to characterize the beneficiaries of private health plans who underwent hemodialysis in the SUS, from 2012 to 2019, in relation to: gender, age group, region of residence, characteristics of the private health plans and the operators and of the care provided to them. The characteristics of the private health plans and the modality of the operators of the beneficiaries where then compared with data of the other beneficiaries in Brazil. An individual-centered database was constructed based on data from the Brazilian National Supplementary Health Agency (ANS); information on beneficiaries in Brazil was consulted in Brazilian Health Informatics Department (DATASUS). Frequency distributions were used to summarize the data, standardization by age and sex for characteristics of the private health plans and modality of the operators and ratio to compare frequencies. A total of 31,941 beneficiaries underwent hemodialysis in the SUS, 11,147 (34.9%) outside their municipality of residence, and 6,423 (20.11%) used the SUS for 25 months or more. When compared with other beneficiaries in Brazil, those who underwent hemodialysis in the SUS were more frequently linked to old private health plans (ratio, r = 2.41), collective by adherence (r = 1.76), individual/family (r = 1.36), outpatient (r = 4.66), municipal (r = 3.88), and/or philanthropic (r = 7.32). Private health plans with restrictive characteristics may have hindered the access of beneficiaries who performed hemodialysis in SUS to the networks of their operators and have represented one more among the factors that may have influenced the use of SUS by those beneficiaries, even with coverage provided for in their contracts.

## Abstract (es)

El resarcimiento al Sistema Único de Salud (SUS) es la interfaz más visible de la relación público-privada y su análisis puede ampliar el conocimiento sobre el uso del SUS por el sector suplementario. El estudio tuvo como objetivo caracterizar a los beneficiarios de planes privados de salud que realizaron hemodiálisis en el SUS, entre 2012 y 2019, con relación a: género, rango de edad, región de residencia, características de los planes privados de salud y de los operadores y a la asistencia prestada a ellos; y comparar características de los planes privados de salud y modalidad de los operadores de aquellos beneficiarios con datos de los demás beneficiarios de Brasil. Se construyó una base centrada en el individuo a partir de datos de la Agencia Nacional de Salud Suplementaria (ANS); las informaciones sobre los beneficiarios en Brasil fueron consultadas en el Departamento de Informática del SUS (DATASUS). Se utilizaron distribuciones de frecuencia para resumir los datos, la estandarización por edad y género para las características de los planes privados de salud y la modalidad de los operadores y la relación para comparar frecuencias. Un total de 31.941 beneficiarios se sometieron a hemodiálisis en el SUS, 11.147 (34,9 %) fuera de su municipio de residencia y 6.423 (20,11 %) utilizaron el SUS por 25 meses o más. Comparados a los demás beneficiarios de Brasil, los beneficiarios que se sometieron a hemodiálisis en el SUS estaban vinculados más frecuentemente a planes privados de salud antiguos (razón, r = 2,41), colectivos por adhesión (r = 1,76), individuales/familiares (r = 1,36), ambulatorios (r = 4,66), municipales (r = 3,88) y/o a filantropías (r = 7,32). Planes privados de salud con características restrictivas pueden haber dificultado el acceso de los beneficiarios que realizaron hemodiálisis en el SUS a las redes de sus operadores y pueden haber representado un factor más entre los que pueden haber influido en el uso del SUS por aquellos beneficiarios, incluso con cobertura prevista en sus contratos.

## Introdução

O acesso aos serviços públicos no sistema de saúde brasileiro é universal, para todo cidadão. Porém, o setor privado oferta os mesmos serviços realizados pelo setor público ^1^^,^^2^, esse último com assistência mais ampla e integral em relação ao primeiro, gerando uma cobertura duplicada para 25% da população, vinculada a planos privados de saúde ^3^. Assim, beneficiários da saúde suplementar utilizam o Sistema Único de Saúde (SUS) para realizarem alguns procedimentos, mesmo aqueles previstos em seus contratos. Grande parte dessa utilização do SUS é para a assistência ambulatorial de alto custo/complexidade, aumentando a iniquidade na oferta, no acesso e no uso desses serviços ^1^^,^^4^^,^^5^^,^^6^^,^^7^^,^^8^, além de ampliar o lucro das operadoras às expensas de recursos sociais, quando estas se responsabilizam por serviços que acabam sendo prestados pelo SUS ^9^^,^^10^.

Há, desde a década de 1970, previsão para ressarcimento ao erário da assistência prestada a beneficiários de planos privados de saúde, apesar de não ter apresentado registros de restituição dos valores devidos até a *Lei nº 9.656/1998*^4^^,^^11^^,^^12^. Somente a partir de dezembro de 1999 as operadoras começaram a ressarcir valores de atendimentos realizados no SUS e previstos nos planos privados de saúde ^13^, os quais estão disponíveis no site da Agência Nacional de Saúde Suplementar (ANS) ^11^ e no Tabnet do Departamento de Informática do SUS (DATASUS) ^3^.

O ressarcimento ao SUS é a interface mais visível entre os setores público e suplementar de saúde ^13^, e gera dados relevantes à compreensão da utilização do SUS pelos beneficiários de planos privados de saúde ^14^. A assistência que deve ser ressarcida ao SUS é identificada por meio do relacionamento entre o Sistema de Informações de Beneficiários (SIB), da ANS, e os sistemas de informações ambulatorial e de internação do SUS ^13^.

A integração de bases da saúde é uma alternativa para organizar a informação por paciente ^15^, e existe a necessidade de se conhecer o ressarcimento *per capita* dos atendimentos realizados no SUS a beneficiários de planos privados de saúde, uma vez que representa um caminho para uma regulação mais efetiva do mercado suplementar ^16^. Porém o fluxo de beneficiários para o SUS é um tema pouco explorado ^17^. Os estudos até então realizados se estruturaram principalmente no processo administrativo para ressarcimento ao SUS ^13^^,^^14^^,^^18^, o que dificulta realizar inferências sobre o beneficiário que foi atendido pelo sistema ^19^^,^^20^.

A escassez de pesquisas sobre o beneficiário de plano privado de saúde que utiliza o SUS é ainda maior em relação a procedimentos ambulatoriais de alta complexidade, pois a cobrança dessa assistência é recente, desde 2015, retroativa aos atendimentos ocorridos desde 2012 ^13^. Entre os atendimentos ambulatoriais identificados para ressarcimento ao SUS, a hemodiálise sempre foi destaque, tanto em quantidade como no volume financeiro mobilizado ^11^, ensejando uma análise mais aprofundada dessas informações ^10^.

Além disso, a doença renal crônica é um problema de saúde pública mundial. A incidência e a prevalência de pacientes em falência funcional renal que precisam de diálise são crescentes ^21^^,^^22^^,^^23^, e os custos são altos e dependentes de financiamento público ^1^^,^^21^^,^^24^. Desse modo, registros confiáveis e disponíveis sobre pacientes que realizam diálise são fundamentais ao planejamento e à racionalização de recursos econômicos ^21^^,^^25^.

Desde 1999, a Sociedade Brasileira de Nefrologia realiza inquéritos anuais sobre pacientes em diálise, sob financiamento público e privado. Porém a adesão das unidades de diálise ao censo é voluntária, e a subnotificação foi crescente na última década ^21^^,^^22^. Existem outras iniciativas para individualizar os dados nacionais sobre diálises no SUS ^15^^,^^26^^,^^27^, mas sem diferenciar pacientes que também eram beneficiários de planos privados de saúde.

Neste contexto, nosso estudo objetivou caracterizar os beneficiários de planos privados de saúde que realizaram hemodiálise no SUS entre 2012 e 2019, dividindo-os em relação ao sexo, à faixa etária, à região de residência, às características dos planos privados de saúde e das operadoras, e à assistência prestada a eles. Adicionalmente, comparou-se as características dos planos privados de saúde e as modalidades das operadoras daqueles beneficiários com as informações dos demais beneficiários do Brasil.

## Métodos

Estudo transversal, descritivo, quantitativo, utilizando dados não públicos disponibilizados pela ANS. Foram elegíveis todos os beneficiários de planos privados de saúde com doença renal crônica (Classificação Internacional de Doenças, 10ª revisão - CID-10 N180) que realizaram no SUS pelo menos uma hemodiálise (máximo três sessões por semana, 0305010107 no Sistema de Gerenciamento da Tabela de Procedimentos do SUS), e que tiveram essa assistência identificada para ressarcimento, entre 1º de abril de 2012 e 31 de dezembro de 2019. Não utilizamos outros tipos de diálise (sorologias positivas, pediátrica, excepcionalidade e peritoneal) porque representavam menos de 4% dos atendimentos identificados para ressarcimento ao SUS no período estudado.

**Figura 1:**
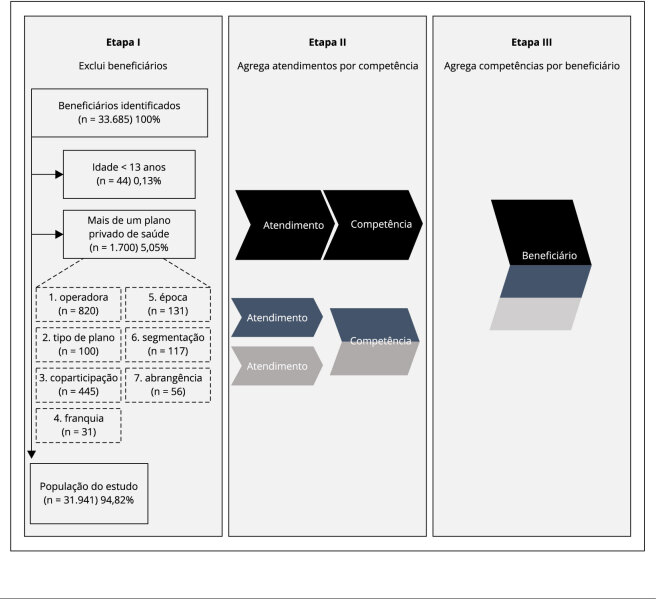
Etapas para construir a base de dados centrada no beneficiário de plano privado de saúde que realizou hemodiálise no Sistema Único de Saúde (SUS), entre 2012 e 2019.

O banco disponibilizado estava organizado com foco nos atendimentos e foi adaptado em três etapas, conforme esquematizado na Figura 1.

Na etapa I, excluímos os beneficiários com idade menor do que 13 anos, uma vez que podem ter sido registrados erroneamente, pois a hemodiálise 0305010107 está vinculada a pacientes a partir dessa idade ^3^. Excluímos também beneficiários com mais de um plano privado de saúde ao longo do período estudado, porque representavam apenas 5,05% da amostra e complexificariam a análise. Na etapa II, as variáveis dos atendimentos foram agregadas por competência. Na etapa III, as informações de todas as competências de cada beneficiário foram agregadas, constituindo a base de dados centrada no beneficiário.

As variáveis foram classificadas em características do plano privado de saúde, da operadora e da assistência prestada, além das sociodemográficas: sexo (masculino; feminino), idade (13-19 anos; 20-44 anos; 45-64 anos; 65-74 anos; ≥ 75 anos), região de residência (região geográfica; Unidade Federativa - UF; Região Metropolitana da capital e interior; capital e interior para Mato Grosso do Sul e Acre; e Distrito Federal ) ^28^.

As variáveis dos planos privados de saúde foram época da contratação (antigo ou novo: anterior ou não à *Lei nº 9.656/1998*), tipo de plano (individual/familiar; coletivo empresarial ou coletivo por adesão), abrangência geográfica da cobertura assistencial (nacional; grupo de estados; estadual; grupo de municípios ou municipal), segmentação assistencial (referência: ambulatorial+hospitalar+obstetrícia; ambulatorial: ambulatorial, com/sem odontologia; ambulatorial+hospitalar: ambulatorial+hospitalar, com/sem obstetrícia e/ou odontologia; ou hospitalar: hospitalar com/sem obstetrícia e/ou odontologia), franquia (sim ou não) e coparticipação (sim ou não). As variáveis da operadora foram: modalidade (medicina de grupo; cooperativa; seguradora; autogestão ou filantropia) e porte (pequeno: ˂ 20 mil beneficiários; médio: 20-100 mil beneficiários; ou grande: ≥ 100 mil beneficiários).

As variáveis da assistência prestada foram: natureza da organização do prestador ^28^ (administração pública; entidade empresarial ou entidade sem fins lucrativos), região da assistência (município de residência do beneficiário; outro município na mesma UF; outra UF na mesma região geográfica; ou outra região geográfica), tempo total de utilização do SUS (competências/meses de assistência: ≤ 3; 4-6; 7-9; 10-12; 13-15; 16-18; 19-21; 22-24; 25-36; 37-48; 49-60; ≥ 61), número de sessões de hemodiálise e valor do atendimento (em reais, acrescido 50% de valoração do ressarcimento). A categoria “não informado” foi incluída às variáveis com dados faltantes.

A proporção (por 10 mil) de beneficiários que realizaram hemodiálise no SUS foi apresentada segundo região geográfica, UF e região da UF de residência. Para diminuir o viés de comparação ^29^, e, na falta de informações sobre beneficiários que realizaram hemodiálise no setor suplementar ^3^^,^^11^, as distribuições de frequências brutas dos beneficiários que realizaram hemodiálise no SUS, segundo época do plano, tipo de plano, abrangência geográfica da cobertura assistencial, segmentação assistencial e modalidade da operadora, foram padronizadas por idade e sexo através do método de ajuste direto ^29^. Utilizou-se a estrutura etária e por sexo dos demais beneficiários do Brasil (média do total de beneficiários de planos privados de saúde de assistência médica do país entre 2012 e 2019, excluídos os beneficiários que realizaram hemodiálise no SUS naquele período). Os dados dos beneficiários do Brasil foram extraídos da interface Saúde Suplementar/ANS do Tabnet do DATASUS ^3^, que não disponibiliza informações de beneficiários segundo franquia, coparticipação e porte da operadora, o que impossibilitou comparar essas características com dados da população estudada.

Utilizamos distribuições de frequências para resumir os dados das variáveis categóricas e medidas de tendência central e de variabilidade para variáveis quantitativas. Para comparar as frequências, foi utilizada a razão entre elas. A análise foi realizada no software estatístico R, versão 4.1.0 (http://www.r-project.org). Este estudo não precisou ser apreciado por comitê de ética, porque utilizou dados secundários anonimizados, e os resultados foram apresentados de forma agregada, sem possibilidade de identificação individual, conforme previsto na *Resolução nº 510*, de 7 de abril de 2016 ^30^.

## Resultados

A distribuição anual dos 31.941 beneficiários de planos privados de saúde que realizaram hemodiálise no SUS foi de 26,57% (2012), 11,4% (2013), 15,13% (2014), 6,68% (2015), 13,06% (2016), 8,98% (2017), 8,99% (2018) e 9,17% (2019). A maioria era do sexo masculino (60,19%) e estava na faixa de 45-64 anos (41,78%), seguida de beneficiários com 65 anos ou mais (29,82%). O grupo feminino era, em mediana, três anos mais jovem e com variabilidade maior nas idades (Tabela 1).

**Tabela 1:** Distribuição dos beneficiários de planos privados de saúde que realizaram hemodiálise no Sistema Único de Saúde (SUS) entre 2012 e 2019, segundo sexo e idade.

Variável	Masculino		Feminino		Total	
	n	%	n	%	n	%
Idade (anos)						
Média [desvio padrão]	55,45 [16,29]		53,18 [17,36]		54,55 [16,76]	
Mediana [intervalo interquartil]	57 [44:67]		54 [40:66]		55 [42:67]	
Mínima	13		13		13	
Máxima	98		97		98	
Faixa etária (anos)						
13-19	203	1,06	182	1,43	385	1,21
20-44	4.725	24,58	3.960	31,14	8.685	27,19
45-64	8.385	43,62	4.961	39,01	13.346	41,78
65-74	3.477	18,09	2.049	16,11	5.526	17,30
≥ 75	2.434	12,66	1.565	12,31	3.999	12,52
Total	19.224	100,00	12.717	100,00	31.941	100,00

A maior parte dos beneficiários que realizaram hemodiálise no SUS residia na Região Sudeste, na UF de São Paulo. Em geral, se concentraram nas regiões metropolitanas das capitais (19.579/31.941 = 61,29%, incluído Distrito Federal), principalmente no Amazonas (268/272 = 98,53%), Amapá (130/133 = 97,74%), Roraima (43/44 = 97,73%) e Acre (76/81 = 93,83%). Por outro lado, nas seguintes UF a maior parte dos beneficiários residia no interior: Santa Catarina (690/778 = 88,69%), Minas Gerais (3.178/5.108 = 62,22%), Paraná (624/1.062 = 58,76%), Mato Grosso do Sul (183/316 = 57,91%) e Paraíba (172/297 = 57,91%). A proporção de beneficiários que realizaram hemodiálise no SUS foi próxima entre as regiões, sendo maior no Nordeste e menor no Sul. Entre as UF e respectivas regiões metropolitanas essa proporção foi discrepante, sendo a menor constatada na Região Metropolitana de Florianópolis (menos da metade da proporção nacional) e a maior na Região Metropolitana do Amapá (mais de três vezes a proporção nacional) (Tabela 2).

**Tabela 2:** Distribuição dos beneficiários de planos privados de saúde que realizaram hemodiálise no Sistema Único de Saúde (SUS) e dos beneficiários do Brasil entre 2012 e 2019, segundo região do país, Unidade Federativa (UF) e região da UF de residência.

Região	Beneficiários que realizaram hemodiálise no SUS		Beneficiários do Brasil *	Proporção ** (por 10 mil beneficiários)
	n	%	n	
Sudeste	20.196	63,23	25.851.611	7,81
São Paulo	9.696	30,36	15.412.794	6,29
Região Metropolitana	6.095	19,08	8.499.390	7,17
Interior	3.601	11,27	6.913.404	5,21
Minas Gerais	5.108	15,99	4.506.404	11,34
Região Metropolitana de Belo Horizonte	1.930	6,04	1.826.661	10,57
Interior	3.178	9,95	2.679.743	11,87
Rio de Janeiro	4.268	13,36	4.954.818	8,61
Região Metropolitana do Rio de Janeiro	3.648	11,42	4.009.695	9,10
Interior	620	1,94	945.123	6,56
Espírito Santo	1.124	3,52	977.595	11,50
Região Metropolitana da Grande Vitória	767	2,40	677.422	11,32
Interior	357	1,12	300.173	11,89
Nordeste	4.893	15,32	5.548.668	8,82
Bahia	1.220	3,82	1.374.375	8,88
Região Metropolitana de Salvador	720	2,25	870.560	8,27
Interior	500	1,57	503.815	9,92
Ceará	937	2,93	1.022.054	9,17
Região Metropolitana de Fortaleza	798	2,50	889.958	8,97
Interior	139	0,44	132.096	10,53
Pernambuco	884	2,77	1.185.626	7,46
Região Metropolitana do Recife	684	2,14	944.692	7,24
Interior	200	0,63	240.934	8,30
Rio Grande do Norte	443	1,39	431.050	10,28
Região Metropolitana do Natal	288	0,90	335.635	8,58
Interior	155	0,49	95.415	16,25
Sergipe	331	1,04	258.080	12,83
Região Metropolitana de Aracaju	271	0,85	216.718	12,50
Interior	60	0,19	41.362	14,51
Paraíba	297	0,93	342.199	8,68
Região Metropolitana de João Pessoa	125	0,39	222.751	5,61
Interior	172	0,54	119.448	14,40
Piauí	274	0,86	232.842	11,77
Região Integrada de Desenvolvimento da Grande Teresina ***	196	0,61	183.758	10,67
Interior	78	0,24	49.084	15,90
Maranhão	270	0,85	382.782	7,05
Região Metropolitana da Grande São Luís	185	0,58	277.677	6,66
Interior	85	0,27	105.106	8,09
Alagoas	237	0,74	319.660	7,41
Região Metropolitana de Maceió	182	0,57	253.740	7,17
Interior	55	0,17	65.919	8,34
Sul	3.720	11,65	5.989.576	6,21
Rio Grande do Sul	1.880	5,89	2.325.996	8,09
Região Metropolitana de Porto Alegre	1.035	3,24	1.242.252	8,33
Interior	845	2,65	1.083.744	7,80
Paraná	1.062	3,32	2.383.789	4,46
Região Metropolitana de Curitiba	438	1,37	1.164.821	3,76
Interior	624	1,95	1.218.968	5,12
Santa Catarina	778	2,44	1.279.791	6,08
Região Metropolitana de Florianópolis	88	0,28	270.198	3,26
Interior	690	2,16	1.009.593	6,84
Centro-oeste	1.863	5,83	2.603.408	7,16
Goiás	751	2,35	905.338	8,30
Região Metropolitana de Goiânia	377	1,18	497.966	7,57
Interior	374	1,17	407.372	9,18
Mato Grosso	371	1,16	446.238	8,32
Região Metropolitana do Vale do Rio Cuiabá	222	0,70	221.286	10,03
Interior	149	0,47	224.952	6,63
Mato Grosso do Sul ^#^	316	0,99	489.422	6,46
Campo Grande	133	0,42	209.258	6,36
Interior	183	0,57	280.164	6,53
Distrito Federal	425	1,33	762.410	5,57
Norte	1.228	3,84	1.466.778	8,37
Pará	367	1,15	669.965	5,48
Região Metropolitana de Belém	278	0,87	441.184	6,30
Interior	89	0,28	228.781	3,89
Rondônia	287	0,90	149.609	19,18
Região Metropolitana de Porto Velho	155	0,49	84.916	18,25
Interior	132	0,41	64.693	20,40
Amazonas	272	0,85	444.722	6,12
Região Metropolitana de Manaus	268	0,84	436.433	6,14
Interior	4	0,01	8.289	4,83
Amapá	133	0,42	57.720	23,04
Região Metropolitana de Macapá	130	0,41	53.915	24,11
Interior	3	0,01	3.805	7,89
Acre ^#^	81	0,25	36.802	22,00
Rio Branco	76	0,24	33.257	22,85
Interior	5	0,02	3.546	14,05
Roraima	44	0,14	24.538	17,93
Região Metropolitana de Boa Vista	43	0,13	24.067	17,87
Interior	1	0,00	471	21,33
Tocantins	44	0,14	83.422	5,27
Região Metropolitana de Palmas	22	0,07	48.486	4,54
Interior	22	0,07	34.936	6,30
Não informado	41	0,13	22.652	18,10
Total	31.941	100,00	41.482.693	7,70

Nota: o banco de dados do SUS disponibiliza informações de faixa etária dos beneficiários segundo intervalos de cinco anos, o que impossibilitou utilizar, para os beneficiários do Brasil, o mesmo recorte de idade da população deste estudo, ou seja, a partir de 13 anos. Por esse motivo, utilizamos para eles a idade mínima de 10 anos. No entanto, os beneficiários com idade inferior a 13 anos que realizaram hemodiálise no SUS quantificaram apenas 0,13% das exclusões utilizadas como critério para chegar à população utilizada nessa pesquisa.

* Distribuição dos beneficiários do Brasil vinculados a planos privados de assistência médica com idade ≥ 10 anos;

** Proporção de beneficiários de planos privados de saúde que realizaram hemodiálise no SUS segundo a região;

*** Região Integrada de Desenvolvimento da Grande Teresina: Região Integrada de Desenvolvimento entre Piauí e Maranhão. Para este estudo foram incluídos apenas beneficiários residentes no Piauí;

^#^ A UF não possui regiões metropolitanas.

Do total de beneficiários, 34,9% foram assistidos fora de seu município de residência, e 95,54% realizaram hemodiálise em prestadores privados. A maior parte utilizou o SUS por até três meses, 39,1% por mais de um ano e 20,11% por 25 meses ou mais. Os beneficiários se concentravam em operadoras de grande porte e em contratos sem previsão de franquia e/ou coparticipação (Tabela 3).

**Tabela 3:** Distribuição dos beneficiários de planos privados de saúde que realizaram hemodiálise no Sistema Único de Saúde (SUS) entre 2012 e 2019, segundo variáveis da assistência prestada, porte da operadora e existência de coparticipação/franquia no contrato (N = 31.941).

Variável	n	%
Tempo total de utilização do SUS (meses)		
≤ 3	9.145	28,63
4-6	4.812	15,07
7-9	3.090	9,67
10-12	2.404	7,53
13-15	1.879	5,88
16-18	1.737	5,44
19-21	1.347	4,22
22-24	1.104	3,46
≥ 25	6.423	20,11
25-36	2.931	9,18
37-48	1.700	5,32
49-60	769	2,41
≥ 61	1.023	3,20
Região da assistência		
Município de residência	20.285	63,51
Outro município na mesma UF	9.128	28,58
Outra UF na mesma região do país	851	2,66
Outra região do país	1.168	3,66
Não informado	509	1,59
Natureza da organização do prestador		
Entidades empresariais	21.707	67,96
Entidades sem fins lucrativos	8.810	27,58
Administração pública	1.394	4,36
Não informado	30	0,09
Porte da operadora		
Grande	20.044	62,75
Médio	8.527	26,70
Pequeno	3.367	10,54
Não informado	3	0,01
Franquia		
Não	29.999	93,92
Sim	1.835	5,74
Não informado	107	0,33
Coparticipação		
Não	17.617	55,15
Sim	14.217	44,51
Não informado	107	0,33

UF: Unidade Federativa.

A mediana do número de sessões de hemodiálise por beneficiário foi 98 (intervalo interquartílico - IIQ = 33:250; mínimo = 1 e máximo = 1.214). A mediana do valor identificado foi R$ 27,97 mil (IIQ = 9,55:71,86; mínimo = R$ 255,80 e máximo = R$ 439,78 mil). Os beneficiários contabilizaram 5,81 milhões de sessões de hemodiálise, a um custo total de R$ 1,67 bilhões nos 93 meses estudados.

A maior parte dos beneficiários que realizaram hemodiálise no SUS estava vinculada a contratos novos, do tipo “coletivo empresariais”, com abrangência geográfica por grupo de municípios e de segmentação assistencial ambulatorial+hospitalar, bem como a operadoras da modalidade “medicina de grupo”. Em relação aos demais beneficiários do Brasil, aqueles que realizaram hemodiálise no SUS estavam vinculados mais frequentemente a contratos antigos (razão, r = 2,41), coletivos por adesão (r = 1,76), individuais/familiares (r = 1,36), ambulatoriais (r = 4,66) e municipais (r = 3,88), e a filantropias (r = 7,32). Assim como os demais beneficiários do Brasil, a maioria dos que realizaram hemodiálise no SUS se concentrava em planos coletivos, mas a frequência de coletivos por adesão era maior (r = 1,76) (Tabela 4).

**Tabela 4:** Distribuição dos beneficiários de planos privados de saúde que realizaram hemodiálise no Sistema Único de Saúde (SUS; N = 31.941) e dos beneficiários do Brasil (N = 41,45 milhões) entre 2012 e 2019, segundo características dos contratos e modalidade das operadoras.

Variável	Beneficiários que realizaram hemodiálise no SUS			Demais beneficiários do Brasil *		Razão entre frequências ** (A/B)
	n	% bruta	% padronizada *** (A)	n	% (B)	
Época da contratação						
Novo	26.776	83,83	44,99	36.970.024	89,19	0,50
Antigo	4.951	15,50	26,06	4.480.728	10,81	2,41
Não informado	214	0,67	28,95	0	0,00	-
Tipo de plano						
Coletivo empresarial	15.724	49,23	30,98	27.595.520	66,57	0,47
Individual ou familiar	10.234	32,04	25,62	7.821.849	18,87	1,36
Coletivo por adesão	5.872	18,38	24,55	5.766.240	13,91	1,76
Não informado	111	0,35	18,85	267.143	0,65	-
Segmentação assistencial						
Ambulatorial+hospitalar	26.378	82,58	22,97	35.170.291	84,85	0,27
Ambulatorial	2.954	9,25	19,72	1.753.733	4,23	4,66
Referência	2.296	7,19	25,32	3.756.603	9,06	2,79
Hospitalar ^#^	202	0,63	16,66	506.611	1,22	13,66
Não informado	111	0,35	15,33	263.514	0,64	-
Abrangência geográfica da cobertura assistencial						
Nacional	7.967	24,94	16,12	17.439.906	42,06	0,38
Grupo de estados	1.878	5,88	20,14	2.351.535	5,67	3,55
Estadual	2.569	8,04	16,52	2.872.404	6,93	2,38
Grupo de municípios	14.510	45,43	16,39	16.963.072	40,93	0,40
Municipal	1.644	5,15	14,62	1.563.273	3,77	3,88
Não informado	3.373	10,56	16,21	260.562	0,64	-
Modalidade da operadora						
Medicina de grupo	13.751	43,05	22,24	15.017.705	36,24	0,61
Cooperativa	11.143	34,89	21,46	15.336.645	37,00	0,58
Autogestão	3.635	11,38	14,90	4.537.304	10,94	1,36
Seguradora	2.361	7,39	24,49	5.601.238	13,51	1,81
Filantropia	1.051	3,29	16,91	957.859	2,31	7,32

* Distribuição da frequência percentual de beneficiários do Brasil vinculados a planos privados de assistência médica, com idade igual ou superior a 10 anos, excluídos os beneficiários que realizaram hemodiálise no SUS;

** Razão entre a distribuição de frequência padronizada de características dos contratos e das operadoras dos beneficiários de planos privados de saúde que realizaram hemodiálise no SUS, e a respectiva distribuição de frequência dos demais beneficiários do Brasil;

*** Distribuição da frequência percentual dos beneficiários de planos privados de saúde que realizaram hemodiálise no SUS, padronizada por idade e por sexo, utilizando a mesma seleção para os demais beneficiários do Brasil vinculados a planos privados de assistência médica, idade ≥ 10 anos;

^#^ Planos de saúde que possuem apenas a segmentação assistencial hospitalar não contemplam atendimentos ambulatoriais. Assim, a existência desse tipo de segmentação na população estudada pode estar relacionada a planos antigos ou a erros de registro nos sistemas de informação da Agência Nacional de Saúde Suplementar (ANS). Entre os beneficiários de planos privados de saúde que realizaram hemodiálise no SUS dentro do período estudado, observou-se que, do total de 202 (0,63%) vinculados a contratos de segmentação hospitalar, 107 (52,97%) eram planos antigos. Todos os demais 95 (47,03%) beneficiários com planos novos utilizaram o SUS por até 3 meses, apenas durante o ano de 2014. Além disso, apenas 3,15% da cobrança de hemodiálise relacionada a esses beneficiários com plano novo e segmentação hospitalar foi considerada procedente pela ANS após análise dos respectivos contratos.

## Discussão

Entre 2012 e 2019, mais de 30 mil beneficiários de planos privados de saúde foram submetidos a 5,8 milhões de sessões de hemodiálise no SUS, e mais de 20% deles utilizaram o SUS por 25 meses ou mais. Se comparados aos demais beneficiários do Brasil, aqueles estavam vinculados mais frequentemente a contratos antigos, coletivos por adesão, individuais/familiares, ambulatoriais, municipais e a filantropias. Planos privados de saúde com características restritivas podem ter dificultado o acesso dos beneficiários que realizaram hemodiálise no SUS às redes de suas operadoras, e terem representado mais um dentre os diversos fatores que podem ter influenciado a utilização da rede pública por aqueles beneficiários em detrimento da cobertura prevista em seus contratos.

A menor quantidade de beneficiários que realizaram hemodiálise no SUS foi registrada em 2015 (6,68%), coincidindo com o início da cobrança para ressarcimento ao SUS dos procedimentos ambulatoriais de alta complexidade ^13^. Esse resultado pode indicar um esforço das operadoras para diminuírem o uso da rede pública por seus beneficiários frente à nova obrigação financeira, refletindo o ressarcimento como uma das ferramentas de regulação do mercado suplementar ao induzir o cumprimento dos contratos. Em 2019, apesar da diminuição do número de beneficiários em 6,66% ^3^, houve incremento de 59,6% na realização de hemodiálise crônica por planos privados de saúde, se comparado a 2014 ^13^. Os inquéritos brasileiros também identificaram aumento de diálises pagas por planos privados de saúde (de 15%, em 2014 ^31^, para 21% em 2019 ^22^).

O sexo e a idade dos beneficiários que realizaram hemodiálise no SUS foram semelhantes aos de pacientes em diálise reportados por outros estudos no Brasil ^25^^,^^27^, e condizentes com a *Pesquisa Nacional de Saúde* de 2013 (PNS 2013) ^32^ e os inquéritos brasileiros de diálise ^21^^,^^22^. Por outro lado, nas PNS 2013 e 2019 não houve diferença significativa por sexo na proporção de pessoas com plano de saúde ^33^^,^^34^. Assim, o perfil de sexo e idade dos beneficiários que realizaram hemodiálise no SUS pode estar mais relacionado à condição de paciente renal crônico do que ao fato de ter um plano privado de saúde.

A maior parte de beneficiários que realizaram hemodiálise no SUS residia no Sudeste, na UF de São Paulo, em regiões com maior concentração de emprego e crescimento econômico ^28^, e que, por isso, concentram a demanda por planos privados de saúde ^33^ e as redes das operadoras ^13^^,^^20^, além de terem recebido maiores investimentos em infraestrutura para terapias renais substitutivas no país ^35^. Era esperado que, na maioria das UF, as regiões metropolitanas das capitais concentrassem os beneficiários que realizaram hemodiálise no SUS, porque essa assistência de alto custo não está disponível em todos os municípios brasileiros. Por outro lado, algumas UF concentraram mais beneficiários no interior do que nas regiões metropolitanas das capitais, o que pode ser reflexo de um processo mais avançado de organização da linha de cuidado à terapia renal substitutiva em redes regionalizadas ^22^^,^^36^, uma política nacional prevista desde 2004 ^35^. Além disso, alguns municípios do interior são importantes polos regionais, ocupando maior destaque socioeconômico e assistencial que as capitais e regiões metropolitanas.

Não existem dados públicos individualizados sobre beneficiários em terapia renal substitutiva no Brasil que permitissem saber quantos desses beneficiários realizaram hemodiálise no SUS. Isso impossibilitou identificar se as desigualdades regionais encontradas nesse estudo refletiam a utilização do SUS pelo setor suplementar, ou se eram reflexo da distribuição daqueles beneficiários nessas regiões. Porém, as maiores proporções de beneficiários que realizaram hemodiálise no SUS estavam nas UF do Nordeste e do Norte, coincidindo com as limitações assistenciais observadas pelas PNS 2013 e 2019, que se mostravam maiores entre pessoas com plano de saúde residentes nessas regiões menos desenvolvidas do país. Nessas PNS, Nordeste e Norte também apresentaram as menores proporções de pessoas que consideraram o plano de saúde bom ou muito bom, sendo o outro extremo ocupado pela Região Sul ^34^. Existem evidências de menor satisfação de beneficiários relacionada a restrições em seus planos privados de saúde, tais como reajustes sem regulação, abrangência geográfica limitada, falhas na cobertura assistencial, custos crescentes das mensalidades e copagamento, em especial para tratamentos de longa duração ^37^, como a hemodiálise crônica. Essas restrições podem levar o beneficiário a utilizar o SUS, mesmo para atendimentos cobertos por seu plano privado de saúde.

Cerca de 28% dos beneficiários utilizaram o SUS por menos de três meses, podendo se relacionar às altas taxas de mortalidade nesse período, às possíveis ocorrências de pacientes renais agudos ^38^^,^^39^, bem como a mecanismos de regulação para garantir o procedimento (prazo máximo: 21 dias úteis) ^13^. No entanto, 39,1% daqueles que realizaram hemodiálise no SUS utilizaram a rede pública por mais de 12 meses sem mudar de plano privado de saúde. Isso sugere que aqueles beneficiários ficaram mais tempo no mesmo plano privado de saúde em relação ao panorama nacional das PNS 2013 e 2019, quando respectivamente 23,5% ^33^ e 22,6% ^40^ das pessoas mantiveram um plano de saúde por 1 ano ou mais sem interrupção. Essa diferença poderia ser explicada porque a troca de planos privados de saúde tem requisitos ^13^ que podem complexificar o processo e/ou significar considerável reajuste de custo, especialmente para a faixa etária de grande parte dos beneficiários que realizaram hemodiálise no SUS, que concentra maiores valores de mensalidade.

Para os 20,11% de beneficiários que realizaram hemodiálise no SUS por 25 meses ou mais, os períodos de utilização não poderiam ser explicados por limitações de carência para realizar hemodiálise quando da contratação de um plano privado de saúde ^13^. Isso porque, para planos novos (83,83% dos beneficiários em nosso estudo), a hemodiálise tem carência máxima de 180 dias, e/ou pode haver cobertura parcial por até 24 meses para doença renal crônica preexistente (planos antigos seguem as regras do contrato). Assim, é possível que aqueles beneficiários estejam sujeitos a mecanismos de regulação para realizar hemodiálise. Além disso, beneficiários cuja hemodiálise era financiada pelo SUS relataram barreiras para utilizarem seu plano privado de saúde em decorrência do alto valor de coparticipação para esse procedimento ^19^.

Neste estudo, para mais da metade dos beneficiários que realizaram hemodiálise no SUS os planos privados de saúde não previam franquia ou coparticipação, coincidindo com a prática de algumas operadoras que, para regularem o acesso aos serviços, utilizaram mais autorização prévia, perícia médica e, em menor proporção, coparticipações ^18^. Mesmo para aqueles 44,51% de beneficiários com contrato coparticipativo, o percentual foi menor em relação aos total de beneficiários do Brasil, que, em 2018, superou 52% ^13^. Ainda, a informação de que existe franquia e/ou coparticipação em um plano privado de saúde não significa que essas se aplicariam à hemodiálise ^13^. Além disso, o paciente que realiza hemodiálise crônica depende dela para manutenção da vida, o que esvazia o sentido de risco moral ^41^, uma das principais justificativas para copagamento em planos privados de saúde ^13^.

O fato de mais de um terço dos beneficiários que realizaram hemodiálise no SUS ter sido assistido fora de seu município de residência pode estar relacionado à política de organização dos serviços de hemodiálise em redes regionalizadas ^36^, o que exige deslocamento para unidades de referência. Porém, em 2019, quase metade dos 805 centros ativos de diálise no Brasil estavam no Sudeste (47%), e as taxas de incidência e prevalência de pacientes em diálise eram desiguais entre estados e regiões, sugerindo limitações no acesso ao tratamento ^21^^,^^22^.

A predominância de prestadores privados em detrimento da rede própria do SUS foi reflexo da política pública de financiamento de hemodiálises no Brasil. Desde a década de 1970, a facilidade para o credenciamento junto à Previdência Social impulsionou a expansão e a consolidação da assistência de alto custo/complexidade predominantemente prestada por serviços privados e alinhada a um mercado monopolizado de equipamentos e insumos para diálises ^35^. Porém, ao contrário do que ocorreu com outros equipamentos de média/alta complexidade/custo, a maior disponibilidade de equipamentos de hemodiálise foi destinada ao SUS, configurando uma dupla-porta de difícil regulação ^42^. Desse modo, em 2019, apesar de quase 25% da população possuir plano privado de saúde médicos ^3^, e de 73% da rede de terapia renal substitutiva ser privada, o financiamento público prevaleceu em 79% das diálises realizadas no Brasil ^22^.

As 5,81 milhões de sessões de hemodiálise que encontramos correspondem a 5,7% do que foi realizado no SUS no mesmo período ^3^, e, mesmo sendo um percentual relativamente pequeno, podem representar aporte importante ao orçamento público ^10^. Por outro lado, durante anos apenas internações foram computadas para ressarcimento ao SUS ^13^, sendo considerado um favorecimento às operadoras ^43^. Neste estudo, inferimos pelo menos R$ 2,66 bilhões não cobrados para este ressarcimento, somente para hemodiálise 0305010107, entre 1º de dezembro de 1999 e 31 de março de 2012, quando as internações já eram cobradas ^13^ (correspondente a cerca de 20% do financiamento SUS para hemodiálise 0305010107 naquele período ^3^). Esse valor poderia ter auxiliado a superar distorções relacionadas à ampliação do lucro das operadoras às expensas de recursos sociais, como também a induzir a prestação de serviços contratados ^10^ para milhares de beneficiários renais crônicos. A diminuição do uso da rede SUS e os valores ressarcidos também poderiam ter sido coadjuvantes para superarem a grande diversidade e desigualdade na oferta e no acesso aos serviços de diálise no Brasil ^21^^,^^22^^,^^25^. Acrescenta-se que a maioria dos pacientes em diálise usa medicamentos contínuos de alto custo, e, como os planos privados de saúde podem excluir alguns deles de sua cobertura ^13^, esse custo também recai sobre o SUS.

Em nosso estudo, 62,75% dos beneficiários eram de operadoras de grande porte, o que pode ser reflexo do mercado suplementar brasileiro, no qual, entre 2012 e 2019, em média 68,65% dos vínculos médico-assistenciais pertenciam a elas ^3^. O fato de estarem vinculados mais frequentemente a planos antigos, em relação aos demais beneficiários do Brasil, pode ter influenciado aqueles que realizaram hemodiálise no SUS a buscarem pela assistência pública. Planos antigos tendem a ser mais restritivos por não se sujeitarem à *Lei nº 9.656/199*8 ^12^ e à cobertura assistencial obrigatória, indispensável para diagnóstico, tratamento e acompanhamento de doenças e eventos em saúde, incluindo a doença renal crônica ^13^.

Assim como os demais beneficiários do Brasil, aqueles que realizaram hemodiálise no SUS tinham, majoritariamente, planos privados de saúde coletivos, o que pode ser resultado da menor disponibilidade de planos individuais/familiares em relação aos coletivos, principalmente os empresariais ^8^. Entre dezembro de 2006 e dezembro de 2021, contratos médico-assistenciais coletivos aumentaram em 51,35%, enquanto individuais/familiares cresceram 0,22% ^3^. Planos privados de saúde coletivos apresentam menores preços iniciais, mas, sem regulação de reajuste, podem igualar e até superar o valor dos individuais/familiares ^8^. Planos coletivos ainda estão sujeitos à rescisão unilateral dos contratos, como também podem estar ligados a uma rede assistencial limitada, pouco resolutiva e, consequentemente, à maior judicialização pelas restrições de coberturas, em especial para procedimentos de maior complexidade e valor ^44^, como a hemodiálise crônica.

Fator agravante é que, para corresponder aos potenciais clientes de planos privados de saúde individuais/familiares, sujeitos à regulação mais incisiva pela ANS ^8^, parte dos planos coletivos podem estar mascarados ao mínimo de dois beneficiários vinculados a uma pessoa jurídica, ou à adesão a associações/entidades sem vínculo representativo com o contratante ^44^. Esses pequenos grupos de beneficiários não têm representatividade e são mais vulneráveis ^13^, podendo estar sujeitos a reajustes mais altos e rede assistencial restrita, comprometendo o acesso aos serviços contratados.

A maior frequência de vínculos coletivos e individuais/familiares dos beneficiários que realizaram hemodiálise no SUS, em relação aos demais beneficiários do Brasil, pode estar relacionada à dificuldade em contratar um plano privado de saúde via empregador. A doença renal crônica e a terapia renal substitutiva afetam negativamente a qualidade de vida dos pacientes ^45^, e, mesmo que não constituam impedimento absoluto à atividade laboral, podem limitá-la ^46^, o que lhes confere o direito de aposentadoria. Além disso, importante proporção das pessoas com plano médico tem o pagamento total (14,5%) ou parcial (30,9%) do seu plano privado de saúde assumido pela empresa ^32^. Assim, planos individuais/familiares e coletivos por adesão, que não encontram auxílio do empregador, tendem a requerer maior participação dos beneficiários no custo das mensalidades, o que pode comprometer ainda mais a renda do paciente renal, já economicamente mais vulnerável em decorrência de sua condição crônica ^23^.

Em relação aos demais beneficiários do Brasil, as frequências de planos privados de saúde ambulatoriais e de abrangência municipal entre os que realizaram hemodiálise no SUS foram maiores. Planos ambulatoriais e municipais configuram a mínima segmentação e cobertura geográfica regulamentadas e são mais baratos ^13^, mas tendem a limitar o acesso aos serviços e a comprometer a integralidade do cuidado, a qualidade e a continuidade da assistência ao beneficiário, com maior peso àqueles portadores de condições crônicas. Estudos apontaram que operadoras, em especial para suprirem a demanda da alta complexidade, direcionaram ao setor público pacientes com planos de preço e qualidade inferiores ^20^^,^^47^.

Por outro lado, o paciente em falência funcional renal, que depende de consultas regulares a especialistas e exames específicos, pode estar contratando um plano privado de saúde para ter acesso mais rápido a esses procedimentos ^48^. Além disso, beneficiários de tais planos podem ter acesso mais oportuno aos exames exigidos para manter ativo o Cadastro Técnico de Rim, em relação àqueles pacientes que não têm um contrato assistencial ^19^.

Nessa perspectiva, é possível que pacientes em terapia renal substitutiva se esforcem para manter um plano privado de saúde a fim de melhorar o acesso aos serviços assistenciais. Um contrato referência (cuja frequência em nosso estudo foi a segunda maior em beneficiários que realizaram hemodiálise no SUS, quando comparados aos demais beneficiários do Brasil) inclui as segmentações ambulatorial+hospitalar+obstetrícia e atendimento integral ilimitado às urgências/emergências após 24 horas da contratação ^13^. Por isso a cobertura de um plano privado de saúde referência é mais ampla, podendo ser uma opção para o paciente em terapia renal substitutiva, mesmo com valor maior da mensalidade em relação aos ambulatoriais ou hospitalares ^13^. Alternativamente, um plano privado de saúde ambulatorial, que é mais barato, já poderia garantir um mínimo para consultas e exames.

Beneficiários que realizaram hemodiálise no SUS foram sete vezes mais frequentes em operadoras filantrópicas quando comparados aos demais beneficiários do Brasil. A oferta de planos privados de saúde por filantropias se organizou em redes assistenciais fragmentadas, uma vez que um mesmo hospital se tornou operadora, além de continuar a prestar assistência ao SUS e a outras empresas do setor suplementar. Como resultado, o acesso dos beneficiários dessas operadoras pode estar restrito a um mínimo de serviços ou a um único estabelecimento hospitalar, muitas vezes localizado em periferias das grandes cidades, ou em municípios menores ^8^^,^^49^. Desse modo, beneficiários de filantropias podem ter planos privados de saúde assistencialmente mais restritivos, em especial para procedimentos de alta complexidade, como a hemodiálise, e, assim, utilizarem mais o SUS.

Ainda sobre as filantropias, ressaltamos que, dentro do período estudado, 21,81% dos equipamentos de hemodiálise no Brasil estavam em entidades sem fins lucrativos ^3^. Logo, é possível que hospitais filantrópicos que comercializam planos privados de saúde, mesmo sendo prestadores de serviços de hemodiálise, estejam contabilizando os gastos e julgando mais econômico ressarcir ao SUS do que custear diretamente a assistência de seus beneficiários. Dentro dessa hipótese, se multiplicaria o subsídio público ao setor privado, uma vez que hospitais filantrópicos, além dos incentivos fiscais, podem contar com repasses públicos para infraestrutura e custeio. Por outro lado, ponderamos que 64,56% dos planos privados de saúde das filantropias são antigos ^3^, não se sujeitando à *Lei nº 9.656/1998*^12^ e à cobertura assistencial obrigatória, incluindo a hemodiálise.

Com base na revisão de literatura realizada, este foi o primeiro estudo a descrever os beneficiários de planos privados de saúde que realizaram hemodiálise no SUS, utilizando uma base centrada no indivíduo, composta pela completude de dados disponíveis quando da solicitação de acesso junto à ANS. Assim, era escassa a literatura sobre beneficiários que utilizaram o SUS, em especial para procedimentos ambulatoriais, cuja cobrança para ressarcimento é recente ^13^, e cujos dados públicos não estão individualizados ^3^. Desse modo, a comparação de nossos resultados com os censos de hemodiálise ^21^^,^^22^ e com as PNS ^33^^,^^34^ deve ser realizada com cautela, uma vez que esses inquéritos utilizam metodologia diferente, e suas populações incluem, além dos beneficiários de planos privados de saúde, dois segmentos não abrangidos pelos dados da ANS: gastos individuais diretos com saúde e planos de assistência a servidores públicos. Além disso, visando diminuir possíveis vieses na comparação entre populações diferentes ^29^, as frequências brutas dos beneficiários que realizaram hemodiálise no SUS foram padronizadas por idade e sexo, utilizando-se a população dos demais beneficiários do Brasil.

Este estudo tem algumas limitações. A hemodiálise exige assistência continuada, o que leva à ocorrência de grande número de atendimentos para um mesmo beneficiário que realizou hemodiálise no SUS. Isso pode ter aumentado a probabilidade de perda de dados para compor a base da assistência identificada para ressarcimento, pois os pares prováveis são encontrados por meio de blocos lógicos, e não por uma identificação unívoca, como o Cadastro de Pessoas Físicas (CPF) ^50^.

Além disso, os beneficiários foram individualizados por meio de identificador único do vínculo contratual do SIB/ANS ^13^. Como uma pessoa pode contratar mais de um plano privado de saúde, sendo cada qual um vínculo diferente, alguns beneficiários dessa pesquisa podem ter sido considerados mais de uma vez. Essa duplicidade de registros pode ser corrigida com investimentos em uma base de dados de identificação unívoca, que atenda às demandas do sistema nacional de saúde brasileiro. A região de residência dos beneficiários é outra limitação, porque a ANS já identificou casos em que o endereço da empresa contratante de plano coletivo, e não o endereço do beneficiário, foi informado indevidamente ^3^^,^^13^. Isso pode resultar em falso aumento de beneficiários no local da empresa em detrimento do local de sua residência.

A predominante dupla-porta dos serviços de diálise no Brasil pode dificultar os mecanismos de auditoria financeira e favorecer que um mesmo atendimento seja cobrado do SUS e da operadora. Por outro lado, beneficiários podem optar por utilizar o SUS, mesmo tendo contratado cobertura para hemodiálise, porque existem serviços de excelência e transporte sanitário públicos, ou mesmo porque o SUS oferece maior garantia de continuidade assistencial diante da incerteza de manter um plano privado de saúde em um país com constantes crises econômicas e tamanhas desigualdades sócio regionais. Nesse contexto, nossos resultados sugerem que planos privados de saúde com características restritivas podem representar mais um dentre os fatores que podem influenciar seus beneficiários a utilizarem a rede pública de hemodiálise em detrimento da cobertura prevista em seus planos privados de saúde.

Por fim, o estudo do ressarcimento ao SUS a partir de uma base centrada no beneficiário de plano privado de saúde ampliou o conhecimento sobre o uso do sistema pelo setor suplementar, para além do enfoque fragmentado sobre atendimentos, valores e processos. Esse tipo de análise tem potencial para lançar luz ao uso do SUS por beneficiários que necessitam de outra assistência ambulatorial contínua, ou utilizam a rede pública para algum tipo de internação recorrente. Porém, a dificuldade de integração de bases públicas da saúde no Brasil é um problema para esses estudos, sendo importante que os órgãos responsáveis aprimorem a divulgação de informações. Reforça-se, assim, a necessidade de apoiar políticas públicas de saúde e orientar políticas de regulação setorial diante da complexa relação entre os setores público e privado de saúde brasileiros.
